# Supplementary material for: Clinical characteristics and persistence of severe acute respiratory coronavirus virus 2 (SARS-CoV-2) IgG antibodies in 4,607 French healthcare workers: Comparison with European countries
Source: Infect Control Hosp Epidemiol. 2020 Nov 4:1–2. doi: 10.1017/ice.2020.1309 (PMC7684179; doi:10.1017/ice.2020.1309)
Supplement: Supplementary file 1 [file icesup.zip › S0899823X20013094sup001.pdf]

CAMBRIDGE JOURNALS

Proof Delivery Form

Infection Control & Hospital Epidemiology

Date of delivery:

Journal and vol/article ref: ice 2001309

Number of pages (not including this page): 2

This proof is sent to you on behalf of Cambridge University Press. Please review the proofs carefully and use the Adobe annotation tools to convey and mark-up edits. Please ensure you answer all queries.

Please EMAIL your annotated PDF within **2** days of receipt to:

Brian Mazeski (iceproduction@cambridge.org)

Authors are strongly advised to read these proofs thoroughly because any errors missed may appear in the final published paper. This will be your ONLY chance to correct your proof. Once published, either online or in print, no further changes can be made.

NOTE: If you have no corrections to make, please also email to authorise publication.

- The proof is sent to you for correction of typographical errors only. Revision of the substance of the text is not permitted, unless discussed with the editor of the journal. Only **one** set of corrections are permitted.
- Please answer carefully any author queries.
- Corrections which do NOT follow journal style will not be accepted.
- A new copy of a figure must be provided if correction of anything other than a typographical error introduced by the typesetter is required.

To order reprints or offprints of your article or a printed copy of the issue, please visit the Cambridge University Reprint Order Center online at: www.sheridan.com/cup/eoc

- If you have problems with the file please email **iceproduction@cambridge.org**

Please note that this pdf is for proof checking purposes only. It should not be distributed to third parties and may not represent the final published version.

Important: you must return any forms included with your proof. We cannot publish your article if you have not returned your signed copyright form

Please do not reply to this email

NOTE - for further information about **Journals Production** please consult our **FAQs** at http://journals.cambridge.org/production_faqs

QUERIES

AQ1: To accommodate published reference citations and indexing, you may wish to add the middle initials for all authors in the byline.

AQ2: The distinction between surnames can be ambiguous, therefore to ensure accurate tagging for indexing purposes online (e.g. for PubMed entries), please check that the highlighted surnames have been correctly identified, that all names are in the correct order and spelt correctly.

AQ3: Please check that affiliations of all the authors and the corresponding author details are correctly set.

AQ4: Please add the author's middle initial to the running head and byline per journal style.

AQ5: Please provide the acknowledgment text.

Infection Control & Hospital Epidemiology (2020), 1–2
doi:10.1017/ice.2020.1309

Letter to the Editor

Clinical characteristics and persistence of severe acute respiratory coronavirus virus 2 (SARS-CoV-2) IgG antibodies in 4,607 French healthcare workers: Comparison with European countries

Chantal Delmas MD[1], Genevieve Plu-Bureau MD, PhD[2], Etienne Canouï MD[3], Luc Mouthon MD, PhD[4] and Jean-Francois Meritet MD[5]

[1]Occupational Health Department, GH Paris Centre – Cochin, APHP, France, [2]Epopee Team Inserm U1153 and Medical Gynecology Unit, GH Paris Centre – Cochin APHP, University of Paris, Paris, France, [3]Antimicrobial Stewardship Team, GH Paris Centre – Cochin, APHP, Paris, France, [4]Internal Medicine Department, GH Paris Centre – Cochin, APHP, University of Paris, Paris, France and [5]Virology Department, GH Paris Centre – Cochin APHP, Paris France

To the Editor—The safety of healthcare workers (HCWs) is a major challenge for healthcare systems. In the course of a severe acute respiratory coronavirus virus 2 (SARS-CoV-2) infection, immunoglobulin G (IgG) antibodies may be detected after a median of 14–24 days (interquartile range [IQR], 10–18) after onset of symptoms.[1]

In France, the coronavirus disease 2019 (COVID-19) pandemic reached a peak on April 7, 2020. HCWs had mobility and flexibility inside the Paris Center university hospital, where there was a cluster in the pandemic. We investigated the prevalence of IgG antibodies against SARS-CoV-2 among all HCWs in this hospital. We also sought to determine the correlation between RT-PCR test and serology and to compare our seroprevalence with that of other European countries.

From May 14, 2020, to June 17, 2020, all HCWs were asked by the occupational health department to participate in serologic screening. The Abbott-Architect test (Abbott Laboratories, Abbott Park, IL) was used to detect IgG anti-SARS-CoV-2. During blood sampling, clinical information was recorded using a standardized self-questionnaire on presented symptoms, comorbidities, and the reverse-transcriptase polymerase chain reaction (RT-PCR) test if one had been previously performed. Blood samples were collected >28 days after the first symptoms from those who were symptomatic.

The seroprevalence and 95% confidence interval were estimated using the Fisher exact method. The *t* test and the χ^2 test were performed to compare quantitative and qualitative variables, respectively. Simple and multivariate logistic regressions were performed to assess risk and symptoms associated with seroprevalence respectively. Statistical analyses were performed using SAS software (SAS Institute, Cary, NC). The local institutional review board approved this study. All subjects participated voluntarily under pseudonyms.

Author for correspondence: Chantal Delmas, E-mail address: chantal.delmas@aphp.fr
Cite this article: Delmas C, *et al.* (2020). Clinical characteristics and persistence of severe acute respiratory coronavirus virus 2 (SARS-CoV-2) IgG antibodies in 4,607 French healthcare workers: Comparison with European countries. *Infection Control & Hospital Epidemiology*, https://doi.org/10.1017/ice.2020.1309

Of 5,021 workers present during the study period, 4,607 (91.8%) were included in the study. The mean age was 41.8 years (SD, 12.6), and 75% were female. Furthermore, 45% were paramedical staff members, 36% were physicians (including medical students), and 19% were in administrative and other professions.

Overall, the prevalence of IgG antibodies was 11.5% (95% confidence interval [CI], 10.6–12.4), and it was significantly higher (ie, 13%) for paramedical staff (P = .04). Age and gender did not differ significantly according to seroprevalence. Furthermore, 5 clinical symptoms were independently associated with positive serology: asthenia, fever, myalgia, ageusia, and anosmia, for which the highest odd ratio (OR) was observed (OR, 11.1; 95% CI, 7.4–16.6). Notably, although anosmia appeared to be the most specific factor, 64.3% of subjects with antibodies did not experience this symptom. The proportion of asymptomatic subjects with a positive serology was 21.4%. When considering comorbidities, positive serology was significantly associated with a lesser prevalence in smokers (OR, 0.41; 95% CI, 0.29–0.58) and a higher prevalence of diabetes (OR, 1.78; 95% CI, 1.04–3.03).

Discordance between RT-PCR and serology

In our study, 19.4% of the study participants had had a RT-PCR. Among individuals with negative a RT-PCR, 51 of 662 (7.7%) had detectable SARS-CoV-2 antibodies, whereas 29 of 233 (12.4%) of RT-PCR–positive participants also had no detectable antibodies. The former result could be explained either by difficulties implementing RT-PCR tests or by the delay between the time of the test and the effective date of infection. For the latter finding, in addition to participants who did not develop antibodies, the time lag between PCR and serology should be mentioned (mean, 64.0 days), which implies that the serology is often realized long after the IgG peak. Indeed, the mean of antibody prevalence in this group (0.28 ± 0.32) was higher than in the negative RT-PCR group (0.05 ± 0.08; $P < .001$). More generally, this group with positive RT-PCR and negative antibody tests had specific characteristics: younger age (38.3 ± 12.8 vs 43.3 ±12.4; $P = .04$), more likely a smoker (31.0% vs 7.4%; $P < 10^{-4}$), and male (37.9% vs 18.1%; $P = .01$) compared with those with positive RT-PCR and positive serology tests.

© The Author(s), 2020. Published by Cambridge University Press on behalf of The Society for Healthcare Epidemiology of America This is an Open Access article, distributed under the terms of the Creative Commons Attribution licence (http://creativecommons.org/licenses/by/4.0/), which permits unrestricted re-use, distribution, and reproduction in any medium, provided the original work is properly cited.

Table 1. Comparison of Seroprevalence IgG in European Countries

Country, First Author	No. of Participants	Prevalence %	95% CI	Date of Blood Test	Population Type
Belgium, Blairon[6]	1,494	1.6	NA	May 25–June 19	4 public hospitals
Belgium, Martin[7]	326	11.0	NA	April 15– May 18	CHU Saint Pierre, Bruxels
UK, Bampoe[3]	200	14.5	9.9–20.1	May 11–June 5	Maternity, London
Germany, Korth[2]	316	1.6	NA	March 25– April 21	Essen Hospital, tertiary-care
Germany, Lackermair[9]	151	2.6	0.8–7.1	April 2–6	Outpatient center, Dachau
Germany, Schmidt[1]	385	2.9	NA	April 20–30	Neurologic clinic
Spain, Garcia-Basteiro[4]	578	7.6	NA	March 28–April 9	Hospital reference, Barcelona
Denmark, Iversen[8]	28,792	2.7	2.5–2.9	April 15–23	Capital region
France, Delmas[a]	4,607	11.5	10.6–12.4	May 14–June 17	Paris Center, university hospital

Note. CI, confidence interval.
[a]Present study.

Comparison with European countries

In our literature review, we retained only studies with IgG antibody testing; we excluded those with IgA or IgM serologies. The 11.5% prevalence of IgG in our HCWs is similar to the reported prevalences in Belgium or the United Kingdom (Table 1). Different protective measures, date of blood screening, and/or population structure in each country could explain the variation in IgG serology from 1.6% reported by Korth et al[2] up to 14.5% reported by Bampoe et al.[3] In our hospital, masks are compulsory, and protective equipment has been available since March 17.

Of 233 HCWs, 29 (12.4%) were RT-PCR positive with no detectable antibodies. This result parallels that of Garcia-Basteiro et al,[4] who also reported 15% of individuals with positive RT-PCR and negative serology. A recent study by Patel et al[5] showed the possibility of decreased antibodies over 60 days, which implies transiently detectable antibodies.

Our study has some limitations. During the lockdown period, some HCWs were isolated at home on a case-by-case basis for reasons of severe personal or familial comorbidities. RT-PCR swab tests were conducted at the time of suspected illness only in symptomatic or in individuals who had had contact with COVID-19 patients. Thus, 902 of 4,607 (19.6%) had this test at the time of onset of symptoms.

The detection of asymptomatic cases by RT-PCR is essential to isolating or avoiding quarantine of HCWs to prevent risk of contamination for vulnerable patients and to reduce the risk of interprofessional staff-to-staff transmission.

To limit virus transmission, we emphasize the necessity of large-scale screening for exposed HCWs, even those who do not present any symptoms. Further investigations are needed to explore negative serology in subjects with positive RT-PCR for understanding population immunity and the potential risks of reinfection and disease in HCWs.

Supplementary material. To view supplementary material for this article, please visit https://doi.org/10.1017/ice.2020.1309

Acknowledgments. AQ5

Financial support. No financial support was provided relevant to this article.

Conflicts of interest. Luc Mouthon reports nonfinancial support from Grifols and Octapharma as well as a grant from LFB and personal fees from Actellion and J&J outside this work. All other authors report no conflicts of interest relevant to this article.

References

1. Schmidt SB, Gruter L, Boltzmann M., Rollnik JD Prevalence of serum IgG antibodies against SARS-CoV-2 among clinic staff. *PLoS One* 2020;15(6). doi: 10.1371/journal.pone.0235417.
2. Korth J, Wilde B, Dolff S, *et al.* SARS-CoV-2–specific antibody detection in healthcare workers in Germany with direct contact to COVID-19 patients. *J Clin Virol* 2020;128:104437.
3. Bampoe S, Lucas DN, Neall G, *et al.* A cross-sectional study of immune seroconversion to SARS-CoV-2 in frontline maternity health professionals. *Anaesthesia* 2020. doi: 10.1111/anae.15229.
4. Garcia-Basteiro AL, Moncunill G, Tortajada M, *et al.* Seroprevalence of antibodies against SARS-CoV-2 among healthcare workers in a large Spanish reference hospital. *Nat Comm* 2020;11. doi: 10.1038/s41467-020-17318-x.
5. Patel MM, Thornsburg NJ, Stubblefield WB, *et al.* Change in antibodies to SARS-CoV-2 over 60 days among healthcare personnel in Nashville, Tennessee. *JAMA Network* 2020. doi: 10.1001/jama.2020.18796.
6. Blairon L, Mokrane S, Wilmet A, Desilly G, *et al.* Large-scale, molecular, and serological SARS-CoV-2 screening of healthcare workers in a 4-site public hospital in Belgium after COVID-19 outbreak. *J Infect* 2020. doi: 10.1016/j.jinf.2020.07.033.
7. Martin C, Montesinos I, Dauby N, *et al.* Dynamics of SARS-CoV-2 RT-PCR positivity and seroprevalence among high-risk healthcare workers and hospital staff. *J Hosp Infect* 2020. doi: 10.1016/j.jhin.2020.06.028.
8. Iversen K, Bundgaard H, Hasselbalch RM, *et al.* Risk of COVID-19 in healthcare workers in Denmark: an observational cohort study. *Lancet* 2020. doi: 10.1016/S1473-3099(20)30589-2.
9. Lackermair K, William F, Grzanna N, *et al.* Infection with SARS-CoV-2 in primary care healthcare workers assessed by antibody testing. *Fam Pract* 2020. doi: 10.1093/fampra/cmaa078.
