# Supplementary material for: Clinical characteristics and persistence of severe acute respiratory coronavirus virus 2 (SARS-CoV-2) IgG antibodies in 4,607 French healthcare workers: Comparison with European countries
Source: Infect Control Hosp Epidemiol. 2020 Nov 4:1–2. doi: 10.1017/ice.2020.1309 (PMC7684179; doi:10.1017/ice.2020.1309)
Supplement: Supplementary file 1 [file icesup.zip › S0899823X20013094sup002.docx]

Table S1. Risk factors and clinical symptoms predictive of SARS-Cov2 antibodies among hospital workers

**A. Risk Factors**

|  | Negative serology  N= 4079 | Positive serology  N=528 | Odd Ratio (CI 95%) |
| --- | --- | --- | --- |
| **Diabetes** | **75 (1.8%)** | **17 (3.2%)** | **1.78 (1.04 – 3.03)** |
| **Smoking** | **646 (15.8%)** | **38 (7.2%)** | **0.41 (0.29 – 0.58)** |
| Atopia | 374 (9.2%) | 42 (8.0%) | 0.86 (0.61 – 1.19) |
| Obesity | 175 (4.3%) | 27 (5.1%) | 1.20 (0.79 – 1.82) |
| Asthma | 259 (6.3%) | 37 (7.0%) | 1.11 (0.78 – 1.59) |
| Hypertension | 273 (6.7%) | 33 (6.3%) | 0.93 (0.64 – 1.35) |

**B. Clinical symptoms**

|  | Negative serology  N= 4079 | Positive serology  N=528 | Odd Ratio (CI 95%)* |
| --- | --- | --- | --- |
| **Anosmia** | **97 (2.4%)** | **231 (43.7%)** | **11.11 (7.45 – 16.58)** |
| **Ageusia** | **107 (2.6%)** | **205 (38.8%)** | **2.22 (1.45 – 3.40)** |
| **Myalgia** | **454 (11.1%)** | **205 (38.8%)** | **1.78 (1.32 – 2.39)** |
| **Fever** | **479 (11.7%)** | **200 (37.9%)** | **1.52 (1.14 – 2.04)** |
| **Asthenia** | **870 (21.3%)** | **292 (55.3%)** | **1.45 (1.09 – 1.93)** |
| Dyspnea | 246 (6.0%) | 126 (23.9%) | 1.42 (0.99 – 2.04) |
| Cough | 726 (17.8%) | 214 (40.5%) | 1.05 (0.79 – 1.40) |
| Rhinitis | 744 (18.2%) | 159 (30.1%) | 0.70 (0.52 – 0.93) |
| Diarrhea | 338 (8.3%) | 121 (22.9%) | 1.20 (0.87 – 1.66) |
| Chest tightness | 263 (6.5%) | 109 (20.6%) | 1.11 (0.77 – 1.60) |
| Headache | 716 (17.6%) | 221 (41.9%) | 0.98 (0.74 – 1.30) |
|  |  |  |  |

*95%CIs of the odds ratios based on multivariable (with all symptoms included in the model) logistic regression analyses.

**Table S2 : Characteristics of subjects according to SARS-Cov2 antibodies and PCR testing**

| **Serology** | **PCR*** | **N (%)** | **Age**  **(mean ± SD)** | **Gender**  **N (% women)** | **Smoking**  **N (%)** | **Comorbidities**  **N (%)** | **Asymptomatic**  **N (%)** |
| --- | --- | --- | --- | --- | --- | --- | --- |
| Negative  (n=4073) | Negative | 611 (13.3) | 42.0 ± 11.9 | 477 (78.1) | 101 (16.5) | 236 (38.6) | 95 (15.5) |
|  | Positive | 29 (0.6) | 38.3 ± 12.8 | 18 (62.1) | 9 (31.0) | 7 (24.1) | 0 (0) |
|  | Not tested | 3433 (74.5) | 41.8 ± 12.7 | 2554 (74.4) | 536 (15.6) | 1012 (29.5) | 2118 (61.7) |
| Positive  (n=527) | Negative | 51 (1.1) | 40.2 ± 11.9 | 40 (78.4) | 6 (11.8) | 26 (51.0) | 4 (7.8) |
|  | Positive | 204 (4.4) | 43.3 ± 12.4 | 167 (81.9) | 15 (7.4) | 69 (33.8) | 4 (2.0) |
|  | Not tested | 272 (5.9) | 41.5 ± 13.2 | 202 (74.3) | 17 (6.2) | 68 (25.0) | 1. 8.6) |

* 7 PCR was undetermined (borderline)
